# Formulation and characterization of physicochemical, functional, morphological, and antioxidant properties of cassava‐based rice analogue

**DOI:** 10.1002/fsn3.2785

**Published:** 2022-02-21

**Authors:** Chiew Yen Liu, Raihan Amani, Syazana Sulaiman, Kaiser Mahmood, Fazilah Ariffin, Abdorreza Mohammadi Nafchi

**Affiliations:** ^1^ Food Technology Division School of Industrial Technology Universiti Sains Malaysia Penang Malaysia; ^2^ Department of Food Science and Technology Damghan Branch, Islamic Azad University Damghan Iran

**Keywords:** artificial rice, cassava leaves, cassava roots, chlorophyll, *Manihot esculenta*, rice analogue

## Abstract

This study aimed to develop a cassava‐based rice analogue with improved nutritional value as an alternative to commercial white rice. The rice analogue formulations (RAFs) were developed by the substitution of modified cassava flour (MOCAF) with rice flour at different ratios as 1:0, 7:3, 5:5, 3:7, and 0:1, followed by the addition of cassava leaves (0, 10, and 20 wt%). The developed rice analogues were evaluated for physicochemical, functional, morphological, and antioxidant properties. The rice analogue containing a 5:5 ratio of rice flour to MOCAF (RAF 2) added with 20% cassava leaves presented the most desirable nutritional composition and functional properties. However, RAF 4 (100% MOCAF with 20% cassava leaves) showed the highest total polyphenol content (TPC) (198.8 mg gallic acid equivalent (GAE)/100 g), total chlorophyll content (TCC) (198 mg/ml), 2,2‐diphenyl‐1‐picrylhydrazyl (DPPH) inhibition (79%), and ferric reducing antioxidant power (FRAP) inhibition (85%). Hence, the addition of MOCAF and cassava leaves improved the nutritional value of cassava‐based rice analogues, which could be a healthy alternative to commercial rice in the daily diet.

## Graphical abstract

Utilization of cassava roots and leaves in cassava‐based rice analogue produces nutrition‐rich rice alternative. Gradual substitution of rice flour with cassava flour, combined with addition of cassava leaf powder, improved nutritional and antioxidant properties of rice analogue. Cassava‐based rice analogue showed potential as an alternative to commercial white rice.

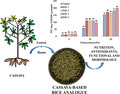

## 1 INTRODUCTION

The improved understanding of health brought the limelight toward a nutritious diet. A healthy diet involves a pattern of food intake that is beneficial or at least possesses no harmful effect on health (Delvarianzadeh et al., 2020; de Ridder et al., 2017). Staple foods are the most important calorie sources that need to be improved in order to maintain good health and control diet‐related ailments (Ghasemi et al., 2022). Thus, the development of functional food such as rice analogue (artificial rice) is a possible option to tackle this issue.

Rice analogue or artificial rice imitates rice and resembles the quality of rice but is made from nonpaddy carbohydrates (Noviasari et al., 2017; Valencia & Purwanto, 2020). Rice analogue suits the dietary needs of a specific person and could improve vigor by delivering various bioactive compounds as well. The main ingredient of rice analogue could be flour obtained from crops such as cassava, sago, corn, and sorghum (Sumardiono et al., 2014). A recent study on rice analogues mainly involved the usage of a variety of flour such as cassava, corn, and sorghum. Rice analogue was made from purple sweet potato flour with zinc fortification, low glycemic index rice analogue was made from corn flour (Kurniawati et al., 2016), and sorghum flour and sago with spices fortification produced rice analogue for diabetic diet (Rasyid et al., 2016). These studies show that rice analogues can be developed from a variety of nonpaddy flour. The cassava plant, which includes the roots and leaves, has potential for this matter.

Cassava is one of the world's most important crops. It is easily grown, rich in carbohydrates, and wholly beneficial (Javadian et al., 2021; Tamimi et al., 2021). Cassava roots contain a high amount of carbohydrates (32%–35%), in which 80% of this component is in starch form with 83% amylopectin and 17% amylose but low amount of lipid (0.1%–0.3%) and protein (0.4%–1.5%) in fresh basis (Montagnac et al., 2009a). In contrast, cassava leaves contain a high amount of protein that ranges between 17.7% and 38.1% (Latif & Müller, 2015) and antioxidants such as polyphenols (16–29 mg/g), vitamin C (1.49–2.81 mg/g), and chlorophyll (565–771 SPAD units) (Santos et al., 2013). In addition, the usage of cassava leaves as a food source could reduce the amount of waste produced by cassava plantations, as it was reported that up to 710 kg of leaves was produced as a by‐product (Howeler, 2012). Therefore, the use of cassava roots and leaves could produce a highly nutritious rice analogue.

Cassava leaves are usually consumed as cooked or boiled vegetables. In Indonesia, they are usually consumed as vegetables, side dishes, and fish balls (Howeler, 2012). In Sierra Leone, cassava leaves are eaten as a rich source of protein, cooked with fish, onions, groundnuts, and capsicums (Latif & Müller, 2015).

Currently, there are very limited studies conducted on the usage of cassava roots and leaves as a source of food. Thus, in this study, rice analogue was developed by substitution of rice flour with modified cassava flour (MOCAF) and by addition of cassava leaves. The physicochemical, functional, and antioxidant properties of cassava‐based rice analogues are evaluated in order to estimate their potential as a functional food. The developed cassava‐based rice analogue would be a suitable alternative to rice and would maximize cassava utilization.

## 2 MATERIALS AND METHODS

### 2.1 Materials

Raw ingredients such as white rice, cassava roots, and leaves were bought locally from Penang, Malaysia. Analytical grade sulfuric acid, sodium hydroxide, sodium carbonate, sodium acetate, ferric chloride, petroleum ether, ammonium solution (28%), potassium iodide, and acetic acid glacial were purchased from Orec (Malaysia). Kjeldahl catalyst tablet, silver nitrate solution, and iodine solution were obtained from Merck (Malaysia). Boric acid, ethanol absolute, methyl spirit, and hydrochloric acid were purchased from Ever Gainful Enterprise (Malaysia). Lactic acid bacteria (BIMO‐CF) starter culture was obtained from AZ Farm (Malaysia). However, amylose standard from potato starch, DPPH reagent, 2,4,6‐Tri(2‐pyridyl)‐s‐triazine (TPTZ), Folin–Ciocalteu's reagent, chlorophyll, and gallic acid were purchased from Sigma‐Aldrich (Malaysia).

### 2.2 Rice analogue preparation

Rice analogue was produced using grounded Jasmine white rice, modified cassava flour (MOCAF), and cassava leaves powder based on the formulation shown in Table 1. Modified cassava flour (MOCAF) of roots and cassava leaves were produced by fermentation for 24 h with lactic acid bacteria and dried at 60°C and 40°C, respectively. Rice analogue was produced using the extrusion method and dried at 60°C.

**TABLE 1:** Rice analogue formulations (RAFs)

Formulation	Ratio of rice flour: MOCAF	Modified cassava flour (MOCAF) (g)	Rice flour (g)	Cassava leaves (g)
Control	1:0	0	100	0
		0	100	10
		0	100	20
RAF 1	7:3	30	70	0
		30	70	10
		30	70	20
RAF 2	5:5	50	50	0
		50	50	10
		50	50	20
RAF 3	3:7	70	30	0
		70	30	10
		70	30	20
RAF 4	0:1	100	0	0
		100	0	10
		100	0	20

Note

Control = Rice analogue with 1:0; RAF 1 = Rice analogue with 7:3; RAF 2 = Rice analogue with 5:5; RAF 3 = Rice analogue with 3:7; RAF 4 = Rice analogue with 0:1. All the ratios are expressed in terms of the ratio of rice flour to modified cassava flour (MOCAF).

### 2.3 Composition analysis

Compositions of rice analogue, which includes crude protein, crude fat, crude fiber, ash, and moisture content, were evaluated by standard methods (AOAC, 2007). The total carbohydrate content was calculated using the difference method as follows:

$$\begin{array}{c} \text{Total carbohydrate (}\%)\\ =100‐\left(\text{moisture}\;\%+\text{ash}\;\%+\text{crude protein}\;\%+\text{crude fat}\;\%\right) \end{array}$$

### 2.4 Amylose contents (%)

Amylose content was analyzed according to a spectrophotometric method and reported in percentage (Juliano, 1971).

### 2.5 Cyanide contents (%)

Cyanide content was determined in accordance with AOAC (Association of Official Analytical Chemists) (915.03) titrimetric method, and the result was given in percentage.

### 2.6 Functional properties

The swelling power and water absorption capacity of RAF were analyzed according to methods described by (Ma et al., 2015). Swelling power and water absorption capacity were estimated as follows:

$$\text{Swelling power}=\frac{\text{Weight of wet precipitate}}{\text{Weight of dried sample}}$$

$$\begin{array}{c} \text{Water absorption capacity}\\ =\frac{\text{Weight of wet precipitate}‐\text{weight of dry precipitate}}{\text{Weight of dried sample}}\times 100 \end{array}$$

### 2.7 Color analysis

Color measurement was made using a colorimeter (CM‐3500d, Konica Minolta, USA), and data were reported in terms of whiteness (L*), redness (a*), and yellowness (b*).

### 2.8 Texture profile analysis

Texture profile analysis (TPA) was conducted according to (Li et al., 2016) by using a TA‐XT2i Texture Analyser (Stable Micro Systems, UK). A 36‐mm aluminum cylinder probe with 5 kg load cell was used. Ten grams of cooked sample was compressed using 70% compression force. Data were reported as hardness, adhesiveness, springiness, cohesiveness, chewiness, gumminess, and resilience.

### 2.9 Total polyphenol content (TPC), total chlorophyll content (TCC), and antioxidant properties

The extraction of bioactive compounds from powdered RAF was done based on the methanol extraction method given by (Hossain et al., 2020). The total polyphenol content (TPC) was determined using Folin–Ciocalteu's reagent (Poonsri et al., 2019), and the results were expressed as gallic acid equivalent (mg GAE/100 g sample). The total chlorophyll content (TCC) was determined by a spectrophotometric method developed by (Ašimović et al., 2016) and the outcome was reported in mg/ml of extract.

The antioxidant activity of RAF extract was measured based on the scavenging activity of 2,2‐diphenyl‐1‐picrylhydrazyl (DPPH) radical according to (Hasan et al., 2009) and the results were reported in inhibition percentage (%). Similarly, ferric reducing antioxidant power (FRAP) assay was performed according to a method described by (Poonsri et al., 2019).

### 2.10 Morphology

Morphology of RAF was analyzed using a field emission scanning electron microscope (FESEM) (Quanta 650 FEG SEM, FEI Technologies Inc., USA). The sample was coated with a thin layer of gold using a Sputter Coater (Q150R, Quorum Technologies Ltd., East Sussex, UK). The structures were observed for longitudinal and cross‐sectional views with 600, 100, and 200× magnification power.

### 2.11 Statistical analysis

Data were collected in triplicate. The data generated were analyzed statistically using a two‐way analysis of variance (ANOVA), and means were compared by Tukey's test (*p* < .05) using SPSS 24.0 (IBM, USA). Pearson's correlation (*p* < .01) was used to determine the correlation of data between all response variables.

## 3 RESULTS AND DISCUSSION

### 3.1 Proximate analysis

The results of moisture, crude protein, crude fat, crude fiber, ash, and carbohydrate contents are displayed in Table 2.

**TABLE 2:** Proximate composition of rice analogue formulation (RAF) based on different ratios of rice flour to modified cassava flour (MOCAF) and by addition of cassava leaves

Formulation	0% cassava leaves	10% cassava leaves	20% cassava leaves
*Moisture (%)*			
Control	6.41 ± 0.27^Aa^	8.36 ± 0.07^ABb^	7.01 ± 0.45^Aa^
RAF 1	6.59 ± 0.36^Aa^	8.15 ± 0.31^Ab^	8.72 ± 0.16^Bb^
RAF 2	6.18 ± 0.25^Aa^	8.88 ± 0.29^BCb^	8.38 ± 0.76^Bb^
RAF 3	8.66 ± 0.21^Ba^	9.49 ± 0.26^Db^	9.58 ± 0.23^Bc^
RAF 4	9.14 ± 0.20^Ba^	9.37 ± 0.10^CDa^	9.04 ± 0.59^Ba^
*Crude proteins (%)*			
Control	9.59 ± 0.25^Ea^	12.67 ± 0.17^Db^	14.97 ± 0.55^Dc^
RAF 1	7.52 ± 0.31^Da^	10.43 ± 0.30^Cb^	12.77 ± 0.26^Cc^
RAF 2	6.16 ± 0.20^Ca^	9.05 ± 0.30^BCb^	10.91 ± 0.20^Bc^
RAF 3	4.78 ± 0.21^Ba^	7.55 ± 0.20^Bb^	8.39 ± 0.23^Ac^
RAF 4	2.03 ± 0.21^Aa^	5.85 ± 0.10^Ab^	8.01 ± 0.50^Ac^
*Crude fat (%)*			
Control	0.49 ± 0.20^Aa^	1.13 ± 0.11^Aa^	2.39 ± 0.52^ABCb^
RAF 1	0.45 ± 0.14^Aa^	0.60 ± 0.28^Aa^	4.27 ± 0.98^Cb^
RAF 2	0.57 ± 0.26^Aa^	0.71 ± 0.30^Aa^	0.73 ± 0.44^Aa^
RAF 3	0.87 ± 0.33^Aa^	3.40 ± 0.53^Bb^	3.19 ± 1.36^BCb^
RAF 4	0.43 ± 0.12^Aa^	0.67 ± 0.06^Aa^	1.85 ± 0.58^Ab^
*Crude fiber (%)*			
Control	1.07 ± 0.12^Aa^	1.65 ± 0.64^Aa^	1.46 ± 0.11^Aa^
RAF 1	1.08 ± 0.76^Aa^	1.95 ± 0.25^Aa^	2.25 ± 0.40^Aba^
RAF 2	1.28 ± 0.36^Aa^	1.99 ± 0.17^Aa^	2.14 ± 0.11^Aba^
RAF 3	1.41 ± 0.78^Aa^	3.27 ± 0.98^Aa^	3.84 ± 1.65^Ba^
RAF 4	4.17 ± 0.45^Ba^	3.06 ± 0.84^Aa^	3.45 ± 0.39^Aba^
*Ash (%)*			
Control	0.40 ± 0.1^Aa^	0.98 ± 0.02^Ab^	1.38 ± 0.05^BCc^
RAF 1	0.47 ± 0.01^Aa^	0.95 ± 0.02^Ab^	1.24 ± 0.02^ABc^
RAF 2	0.43 ± 0.11^Aa^	1.10 ± 0.30^Bb^	1.16 ± 0.20^Ab^
RAF 3	0.61 ± 0.03^Aa^	1.26 ± 0.04^Cb^	1.52 ± 0.05^CDc^
RAF 4	0.85 ± 0.08^Ba^	1.27 ± 0.11^Cb^	1.65 ± 0.0.4^Dc^
*Carbohydrates (%)*			
Control	83.11 ± 0.49	76.85 ± 0.36	74.30 ± 0.67
RAF 1	84.96 ± 0.54	79.87 ± 1.05	72.99 ± 1.22
RAF 2	86.66 ± 0.54	80.25 ± 0.70	78.82 ± 0.56
RAF 3	85.07 ± 0.37	78.30 ± 0.72	77.32 ± 1.19
RAF 4	87.55 ± 0.57	82.82 ± 0.68	79.45 ± 0.28

Note

Mean ± standard deviation with different superscripts column wise (capital alphabets) and row wise (small alphabets) differ significantly at *p* < .05. Control = Rice analogue with 1:0; RAF 1 = Rice analogue with 7:3; RAF 2 = Rice analogue with 5:5; RAF 3 = Rice analogue with 3:7; RAF 4 = Rice analogue with 0:1. All the ratios are expressed in terms of the ratio of rice flour to MOCAF.

Moisture is one of the important parameters which affect the shelf‐life of rice analogue. Moisture content must be controlled well by processing such as drying in order to ensure a longer shelf‐life of rice analogue. Lowering moisture content is effective in preventing mold growth and the proliferation of insects (Los et al., 2018). Fungal growth could happen due to the high respiration rate of rice grain with high retention of moisture content (Atungulu et al., 2016). According to Los et al. (2018), moisture content of dried grain is normally controlled between 10% and 14%. Moisture content was low in all the formulations of RAF and ranged between 6.59% and 9.58% but remained higher than the control. Significant changes were observed for the moisture content of RAF based on the different proportions of MOCAF and cassava leaves. The positive relation between the percentage of cassava leaves and moisture content could be observed, though the trend was not uniform.

The crude protein content of RAF increased when the percentage of cassava leaves addition increased (*p* < .05). The addition of 20% cassava leaves resulted in the highest protein content (Table 2). Cassava leaves are a rich source of protein ranging between 11.8% and 22.7% (Popoola et al., 2019); therefore, a high protein level of cassava leaves could be utilized in the production of RAF. However, without the addition of cassava leaves, rice analogue made from rice flour contains more protein, as compared to cassava flour. Similar to a previous study, rice flour (5.81%) was observed to have more protein than cassava roots (1.32%–1.9%) (Bayata, 2020). On the other hand, the crude fat content of RAF remained between 0.43% and 4.27%, which was higher compared to the control. The samples added with cassava leaves have higher fat content despite the flour type, as no significance was observed between rice and cassava flour‐based rice analogue alone. Cassava leaf possesses higher fat content (3.16%–8.0%) than cassava root (1.38%–3.06%) (Bayata, 2020; Popoola et al., 2019). Hence, the higher fat in RAF 3 with 10% and 20% cassava leaves and RAF 1 with 20% cassava leaves were contributed by the leaves.

Likewise, a significantly higher crude fiber in the RAF (1.08%–4.17%) was noticed compared to the control. The results also showed that the ratio of MOCAF to rice flour has a significant effect on the crude fiber content of RAF. RAF 4 had the highest crude fiber content (4.17%) among all the formulations (Table 2). Besides, RAF with the addition of 20% cassava leaves significantly contains more crude fiber. This implies that besides MOCAF that contains higher fiber compared to rice flour, cassava leaves addition contributes positively to the crude fiber content as well. Fiber plays a major role in improving the overall colon health; thus, a higher fiber content in RAF is nutritionally favorable.

Significant changes in the ash content of RAF were noticed with changing the ratio of MOCAF to rice flour, whereby increased MOCAF substitution resulted in high ash percentage. This implies that MOCAF contains a higher amount of mineral content as compared to rice flour. In line with the current study (Bayata, 2020; Chaudhari et al., 2018) have reported a higher ash content of cassava roots (1.12%–4.33%) than rice (0.30%–0.80%). Likewise, as the percentage of cassava leaves increased, the ash content of RAF also increased. This indicates that ash content was mainly contributed by cassava leaves. Previous studies also presented higher ash content in cassava leaves compared to roots (Alamu et al., 2021; Ferrari et al., 2014).

Carbohydrates are a major fraction of rice and serve as a great energy source for humans by providing glucose after metabolism (Verma & Shukla, 2011); (Chaudhari et al., 2018). The highest carbohydrate content was observed in RAF 4 (Table 2) which shows that RAF can act as an alternative source for staple food in the daily diet. Cassava flour contributes to more carbohydrate content compared to rice flour. However, the addition of cassava leaves made a significant decline in the carbohydrate content of RAF as well. This is reasonable as cassava leaves are rich in protein compared to carbohydrates.

### 3.2 Amylose contents

The amylose content of RAF ranged between 24.1% and 34.1%, which was comparable to control (25.3%–35.0%). (Avaro et al., 2009) categorized the amylose content as low (15%–22%), intermediate (23%–26%), high (27%–30%), and very high (>30%); thus, the RAF could be classified as intermediate, high, and very high in amylose content. Table 3 presents the differences in amylose content of RAF depending on MOCAF to rice flour ratio. The highest amylose content was observed in control fortified with 10% cassava leaves, since it was prepared by Jasmine rice which has high amylose. However, RAF 3 with 10% cassava leaves stood second in terms of amylose content (32.56%), followed by RAF 3 with 20% cassava leaves (31.89%). This implies that higher amylose content could be correlated to the higher ratio of MOCAF in the formulation, and analogous product was reported by (Schmitz et al., 2017). The RAF with intermediate to a high level of amylose is considered healthier, as the onset of type 2 diabetes and obesity could be controlled by the intake of food with high amylose related to its slower breakdown into glucose by amylase. Higher amylose content in rice was proved to slow down the digestion of starch into glucose due to its resistance toward amylase hydrolysis (Syahariza et al., 2013). Thus, RAF, as a healthier staple, can replace white rice for the consumer in the daily diet.

**TABLE 3:** Amylose content, swelling power, and water absorption capacity of rice analogue formulation (RAF) based on different ratios of rice flour to modified cassava flour (MOCAF) and by addition of cassava leaves

Formulation	0% cassava leaves	10% cassava leaves	20% cassava leaves
*Amylose (%)*			
Control	26.56 ± 0.27^ABa^	35.00 ± 0.07^Db^	25.39 ± 0.45^Ba^
RAF 1	24.63 ± 0.36^Aa^	26.35 ± 0.31^Ab^	24.19 ± 0.16^Aa^
RAF 2	34.12 ± 0.25^Cb^	28.59 ± 0.29^Ba^	27.48 ± 0.76^Ca^
RAF 3	31.89 ± 0.21^Ca^	32.56 ± 0.27^Ca^	31.72 ± 0.23^Da^
RAF 4	28.64 ± 0.20^Ba^	28.90 ± 0.10^Ba^	27.71 ± 0.59^Ca^
*Cyanide (%)*			
Control	0^Aa^	0^Aa^	0^Aa^
RAF 1	0^Aa^	0^Aa^	0^Aa^
RAF 2	0^Aa^	2.97 ± 1.29^Bb^	2.65 ± 0^Bb^
RAF 3	2.38 ± 1.03^Ba^	4.17 ± 1.03^Ba^	5.05 ± 1.76^BCa^
RAF 4	2.33 ± 1.01^Ba^	3.83 ± 0.95^Ba^	6.46 ± 1.02^Cb^
*Swelling power (%)*			
Control	1.01 ± 0.00^Aa^	1.02 ± 0.01^Ab^	1.02 ± 0.00^Ab^
RAF 1	1.03 ± 0.00^ABa^	1.03 ± 0.01^Aa^	1.09 ± 0.02^Cb^
RAF 2	1.09 ± 0.02^Db^	1.04 ± 0.01^ABa^	1.04 ± 0.01^ABa^
RAF 3	1.06 ± 0.02^CDa^	1.07 ± 0.01^BCa^	1.07 ± 0.01^BCa^
RAF 4	1.04 ± 0.01^BCa^	1.09 ± 0.02^Cb^	1.09 ± 0.02^Cb^
*Water absorption (%)*			
Control	66.21 ± 6.44^Aa^	120.29 ± 4.61^Aa^	85.10 ± 4.52^Aa^
RAF 1	109.49 ± 3.59^Ba^	155.23 ± 52.51^Ba^	376.20 ± 66.33^Ca^
RAF 2	390.59 ± 36.68^Da^	173.28 ± 14.15^Ba^	197.29 ± 21.99^ABa^
RAF 3	346.15 ± 82.87^Da^	346.3 ± 40.41^Ca^	252.64 ± 25.10^Ba^
RAF 4	172.37 ± 27.54^Ca^	403.31 ± 21.16^Da^	305.60 ± 86.36^BCa^

Note

Mean ± standard deviation with different superscripts column wise (capital alphabets) and row wise (small alphabets) differ significantly at *p* < .05. Control = Rice analogue with 1:0; RAF 1 = Rice analogue with 7:3; RAF 2 = Rice analogue with 5:5; RAF 3 = Rice analogue with 3:7; RAF 4 = Rice analogue with 0:1. All the ratios are expressed in terms of the ratio of rice flour to MOCAF.

### 3.3 Cyanide contents

Both cassava roots and leaves contain cyanide compounds, which are toxic to humans if consumed in an amount higher than 10 ppm (Montagnac et al., 2009b). Cyanide intoxication with symptoms such as headache, dizziness, confusion, and mydriasis can be demonstrated a few minutes after its ingestion (Graham & Traylor, 2018). The cyanide content of RAF ranged between 0 and 6.46 ppm (Table 3). Reduction of cyanide content could be due to the fermentation of MOCAF and leaves for 24 h using lactic acid bacteria and drying at 60°C for 6 and 4 h, respectively. A previous study showed that drying at 60℃ was sufficient and effective in removing 90% of cyanides in cassava chips (Lambri et al., 2013). (Tefera et al., 2014) observed 97% removal of cyanogenic glucosides by fermentation using lactic acid bacteria. Hence, RAF processing was sufficient to reduce the cyanide content of both cassava roots and leaves to the safer limits (<10 ppm). Results showed that an increasing proportion of both MOCAF and cassava leaves could lead to higher cyanide content in RAF.

### 3.4 Functional properties

Swelling power indicates how well starch hydrates in the presence of water and heat during cooking (Olu‐Owolabi et al., 2011). RAF with higher swelling power indicates it can hydrate quickly and therefore requires a shorter cooking time. The swelling power of RAF remained between 1.0 and 1.09, which was significantly higher than the control (Table 3). The ratio of MOCAF to the rice flour and the addition of cassava leaves showed a pronounced impact on the swelling power of RAF. Previous studies suggested a higher swelling power of cassava starch than rice starch (Kusumayanti et al., 2015); (Yu et al., 2012).

Water absorption capacity measures the behavior of starch during gelatinization (Thomas et al., 2014). In parallel to the swelling power, the water absorption capacity of RAF was strongly improved by the addition of MOCAF compared to the control (Table 3). This implies that cassava starch has a better water absorption capacity compared to rice starch. In line with current data, a higher water absorption capacity of cassava starch compared to rice starch has been reported in previous studies, which was correlated with greater size of cassava starch (Ali et al., 2016; Oladunmoye et al., 2014). However, there was no significant difference noticed in the water absorption capacity of RAF added with cassava leaves.

### 3.5 Color analysis

**FIGURE 1:**
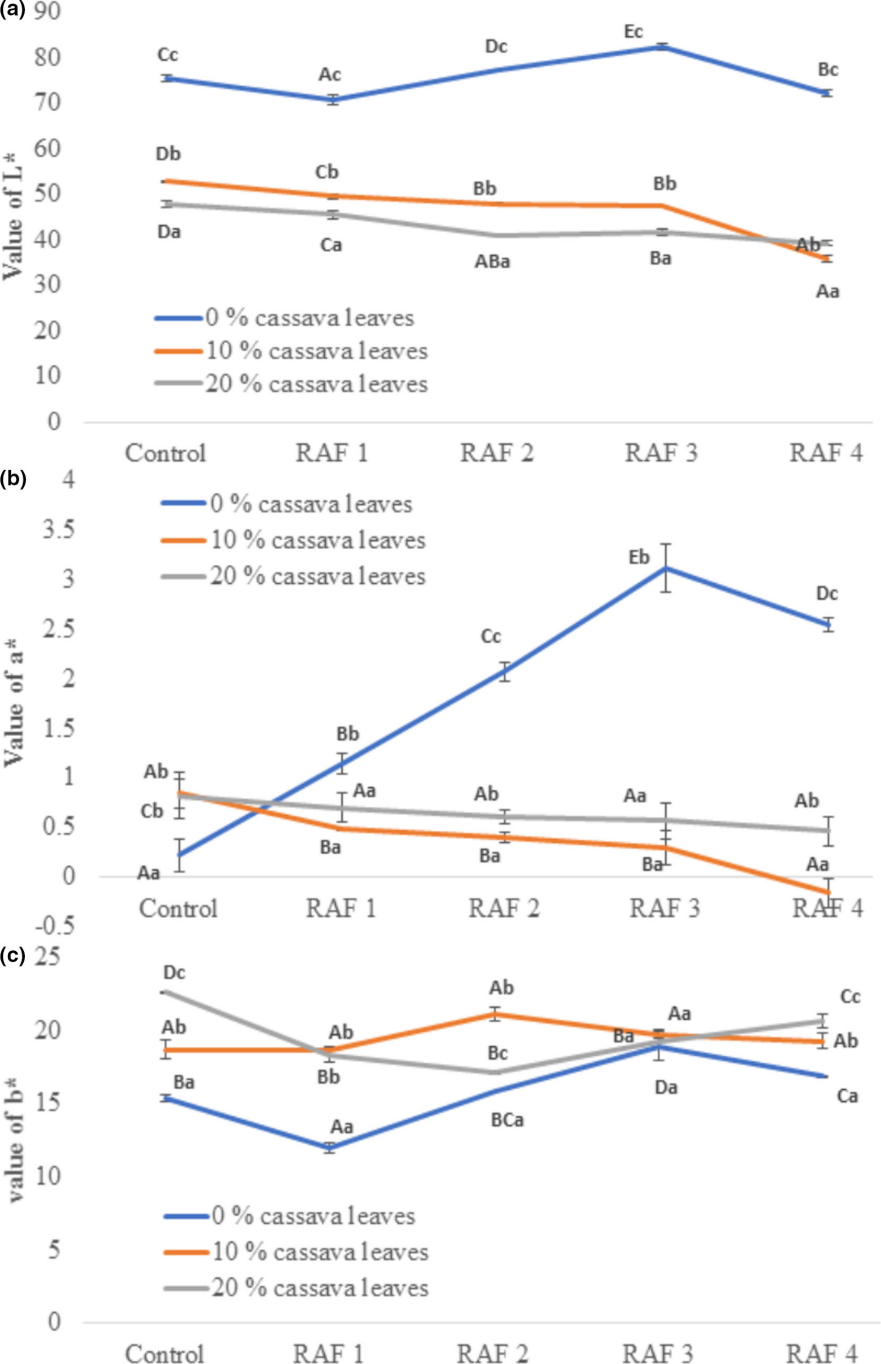
Color parameters as brightness L* (a), redness a* (b), and yellowness b* (c) values of rice analogue formulation (RAF) based on different ratios of rice flour to modified cassava flour (MOCAF) and percentage of leaves addition; Control = Rice analogue with 1:0; RAF 1 = Rice analogue with 7:3; RAF 2 = Rice analogue with 5:5; RAF 3 = Rice analogue with 3:7; RAF 4 = Rice analogue with 0:1. All the ratios are expressed in terms of the ratio of rice flour to MOCAF

Color of food products has a significant impact on purchase intention and consumer acceptance. Based on the appearance, a distinction between color of control and RAF samples added with cassava leaves (0%, 10%, and 20%) can be made clearly (Figure 1). For all the rice analogue formulations, a rise in cassava leaves addition resulted in a greener and darker color.

During food analysis, brightness (L*), redness (a*), and yellowness (b*) are normally analyzed (Markovic et al., 2013). RAF was observed to be brighter than control (high L* value) when no leaves were added. Values of L* and a* decreased, while that of b* increased as the percentage of cassava leaves powder addition increased due to the presence of green pigment in the cassava leaves powder. A similar result was observed previously, which stated that the presence of chlorophyll would lead to a reduction in brightness (Lucas et al., 2018).

### 3.6 Texture profile analysis

The texture profile of rice analogue plays an important role in determining consumer acceptance. The hardness of RAF having MOCAF and cassava leaves ranged between 14.98 and 41.17 N and was comparable to the control (Table 4). The highest hardness was noticed for RAF 1 without the addition of cassava leaves; however, overall, somewhat similar firmness was noticed for all samples with the addition of 20% cassava leaves. This indicates that the hardness of RAF is analogous to that of commercial rice. (Li et al., 2016) stated that amylose content was the major factor that affects the hardness of rice.

**TABLE 4:** Texture profile analysis of the rice analogue formulation (RAF) based on different ratios of modified cassava flour (MOCAF) to rice flour and by addition of cassava leaves

Formulation	0% cassava leaves	10% cassava leaves	20% cassava leaves
*Hardness*			
Control	25.84 ± 5.17^Ba^	29.11 ± 12.76^BCa^	28.57 ± 10.39^Ba^
RAF 1	41.17 ± 24.42^Cc^	27.42 ± 8.79^Bb^	18.78 ± 4.31^Aa^
RAF 2	24.92 ± 8.01^Ba^	17.83 ± 8.14^Aa^	31.08 ± 7.94^Bb^
RAF 3	18.79 ± 5.99^ABa^	26.04 ± 1.15^Bb^	18.88 ± 5.26^Aa^
RAF 4	17.16 ± 6.33^Aab^	14.98 ± 4.81^Aa^	18.93 ± 7.02^Aab^
*Adhesiveness (N.s)*			
Control	−4.59 ± 1.48^Ca^	−6.14 ± 1.64^Ba^	−18.48 ± 2.44^Ab^
RAF 1	−15.10 ± 4.67^Ba^	−8.34 ± 1.26^Bb^	−6.63 ± 1.40^Bb^
RAF 2	−16.88 ± 6.51^Bab^	−14.75 ± 8.14^ABa^	−15.56 ± 1.04^ABa^
RAF 3	−24.10 ± 5.89^Ac^	−20.64 ± 1.85^Aa^	−16.67 ± 6.50^ABab^
RAF 4	−13.07 ± 6.61^Ba^	−16.76 ± 4.67^ABa^	−6.98 ± 0.68^Bb^
*Springiness (mm)*			
Control	0.55 ± 0.23^Aa^	0.44 ± 0.13^Aa^	0.48 ± 0.01^Aa^
RAF 1	0.43 ± 0.12^Aa^	0.50 ± 0.14^Aa^	0.49 ± 0.04^Aa^
RAF 2	0.57 ± 0.02^Aa^	0.60 ± 0.02^Aa^	0.53 ± 0.11^Aa^
RAF 3	0.64 ± 0.14^ABa^	0.53 ± 0.10^Aa^	0.68 ± 0.12^ABa^
RAF 4	0.65 ± 0.07^ABa^	0.69 ± 0.11^ABa^	0.68 ± 0.21^ABa^
*Cohesiveness*			
Control	0.34 ± 0.03^Aa^	0.31 ± 0.01^Aa^	0.40 ± 0.03^Aa^
RAF 1	0.33 ± 0.06^Aa^	0.38 ± 0.05^Aa^	0.41 ± 0.03^Aa^
RAF 2	0.39 ± 0.02^Aa^	0.43 ± 0.02^Aa^	0.44 ± 0.03^Aa^
RAF 3	0.48 ± 0.02^Aa^	0.46 ± 0.08^Aa^	0.44 ± 0.03^Aa^
RAF 4	0.49 ± 0.03^Aa^	0.38 ± 0.05^Aa^	0.47 ± 0.80^Aa^
*Chewiness (g)*			
Control	4.72 ± 1.73^Aa^	4.28 ± 2.54^Aa^	5.51 ± 2.37^ABa^
RAF 1	5.44 ± 2.75^Aa^	5.22 ± 2.44^Aa^	3.64 ± 0.56^Aa^
RAF 2	5.56 ± 2.13^Aa^	4.59 ± 2.20^Aa^	7.18 ± 2.19^ABa^
RAF 3	6.03 ± 3.14^Aa^	6.16 ± 0.50^Aa^	5.93 ± 3.01^ABa^
RAF 4	5.56 ± 2.81^Aa^	4.09 ± 2.37^Aa^	2.83 ± 1.33^Aa^
*Resilience*			
Control	0.13 ± 0.01^Ba^	0.11 ± 0.01^Ba^	0.12 ± 0.01^BCa^
RAF 1	0.10 ± 0.02^Aa^	0.13 ± 0.02^Ba^	0.14 ± 0.01^Ca^
RAF 2	0.09 ± 0.01^Aa^	0.11 ± 0.01^Ba^	0.12 ± 0.01^BCa^
RAF 3	0.09 ± 0.01^Aa^	0.12 ± 0.03^Ba^	0.11 ± 0.01^Ba^
RAF 4	0.16 ± 0.01^Ca^	0.06 ± 0.01^Aa^	0.08 ± 0.01^Aa^

Note

Mean ± standard deviation with different superscripts column wise (capital alphabets) and row wise (small alphabets) differ significantly at *p* < .05. Control = Rice analogue with 1:0; RAF 1 = Rice analogue with 7:3; RAF 2 = Rice analogue with 5:5; RAF 3 = Rice analogue with 3:7; RAF 4 = Rice analogue with 0:1. All the ratios are expressed in terms of the ratio of rice flour to MOCAF.

Adhesiveness refers to the stickiness of rice during decompression or energy required to pull the compressing plunger away from the rice (Kasapis, 2009). Based on Table 4, adhesiveness of RAF ranged between −6.6 and −24.1 N.s, which was lower as compared with control (−4.5 to −18.4 N.s). In a previous report, commercial rice presented adhesiveness between −10.98 and −3.57 N.s (Chen et al., 2017). This implies that RAF had lower adhesiveness and less stickiness as compared to commercial rice. (Yu et al., 2009) observed that the adhesiveness of milled rice has a negative correlation with amylose content. This is because more amylose in high amylose rice tends to leach out and form a coated surface on the rice, hence contributing to less sticky cooked rice (Patindol et al., 2010).

Rice with higher springiness would be more elastic and needs more energy to masticate. There was no significant difference in the springiness of RAF with the addition of different levels of MOCAF and cassava leaves (Table 4). Analogous springiness data were observed for cooked commercial rice (Chen et al., 2017), suggesting that RAF and commercial rice samples are alike in springiness.

Rice with higher cohesiveness would be firmer and tougher to break when compressed between teeth. The addition of MOCAF and cassava leaves did not change the cohesiveness of RAF and remained somewhat comparable to control (Table 4). A comparable outcome was reported in a previous study, whereby springiness was noticed between 0.40 and 0.50 for cooked commercial rice (Chen et al., 2017).

The chewiness is the force that is needed to masticate the rice grain. This implies that rice with higher chewiness would require more energy and time to masticate prior to swallowing. Results showed no differences in chewiness of RAF upon addition of different levels of MOCAF and cassava leaves in the rice flour. This indicates that RAF shared parallel chewiness with commercial rice.

RAF samples presented significant variation in the resilience with changing levels of MOCAF compared to control (Table 4). A similar result was obtained for commercial rice with a range between 0.20 and 0.30 in a previous study (Chen et al., 2017). RAF contains more amylose, resulting in a firmer structure of rice and therefore making it resilient to deformation.

### 3.7 Total phenolics, total chlorophyll content, and antioxidant activity

**FIGURE 2:**
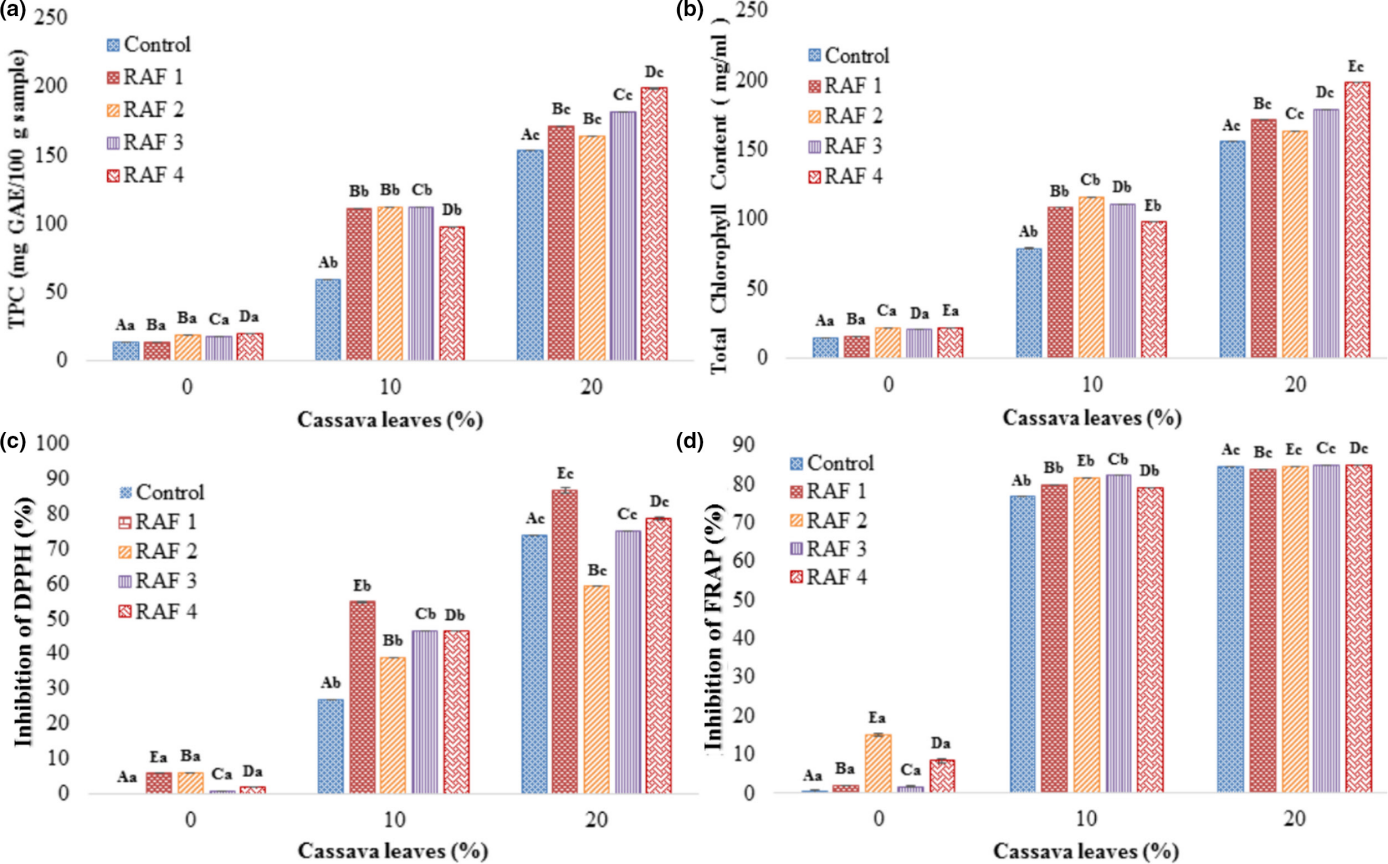
Total phenolic contents (TPCs) (a), total chlorophyll content (TCC) (b), 2,2‐diphenyl‐1‐picrylhydrazyl (DPPH) scavenging (c), and ferric reducing antioxidant power (FRAP) inhibition (d) of rice analogue formulation (RAF) based on different ratios of rice flour to modified cassava flour (MOCAF) and percentage of leaves addition; Control = Rice analogue with 1:0; RAF 1 = Rice analogue with 7:3; RAF 2 = Rice analogue with 5:5; RAF 3 = Rice analogue with 3:7; RAF 4 = Rice analogue with 0:1. All the ratios are expressed in terms of the ratio of rice flour to MOCAF

TPC of RAF significantly (*p* < .05) increased with the substitution of rice flour with MOCAF and addition of cassava leaves (Figure 2a). TPC increased with the addition of cassava leaves, where the highest TPC (199 mg GAE/100 g) was observed in RAF 4 with 20% cassava leaves. (Poonsri et al., 2019) reported a very high TPC (2000 mg GAE/100 g) of rice noodles added with MOCAF and cassava leaves. This difference in TPC might be due to the different substitution ratios of MOCAF in rice flour, as well as by the addition of wheat flour.

Chlorophyll is a green pigment found in cassava leaves and is beneficial to one's health, since it is a natural cleaner of toxins and a good antioxidant that lowers carcinogens and combats antiaging factors (Damayanti & Dahlena, 2020). The TCC of RAF significantly increased with the addition of MOCAF and cassava leaves (Figure 2b). However, TCC was strongly affected by the percentage of cassava leaves added rather than the substitution of MOCAF. This is due to the fact that the cassava leaf contains higher chlorophyll content. RAF samples without cassava leaves showed the lowest amount of TCC (14 mg/ml), while the highest (198 mg/ml) was recorded for RAF 4 with 20% addition of cassava leaves. (Novelina & Hermansyah, 2015) reported that the chlorophyll in cassava leaves increased the antioxidant level in wet noodles. Since plant leaves contain chlorophyll, adding cassava leaves into the food can improve health due to the diverse functional compounds present in the leaves.

DPPH free radical scavenging activity of rice analogue was expressed as percentage inhibition (Figure 2c). DPPH inhibition was significantly increased with the substitution of MOCAF with rice flour and by the addition of cassava leaves. The highest percentage of DPPH inhibition (87%) was observed for RAF 3, while the control sample without cassava leaves recorded the least value. Similar findings were reported previously, stating that the addition of cassava leaf to wet noodles increases the antioxidant activity (Novelina & Hermansyah, 2015; Poonsri et al., 2019). Therefore, a higher substitution ratio of rice flour with MOCAF and the addition of cassava leaves powder in the formulation contribute to higher levels of antioxidants in rice analogue, which might be associated with the number of bioactive compounds available in both ingredients.

Similar to DPPH inhibition, the substitution of MOCAF with rice flour and the addition of cassava leaves in RAF presented better FRAP inhibition, where the highest inhibition (85%) was presented by the RAF 4 having 20% cassava leaves (Figure 2d). The better inhibition of FRAP could be due to the presence of diverse functional phenolics and their derivatives in the cassava leaves. This trend is in agreement with what was reported by (Poonsri et al., 2019), where the highest inhibition was found by rice noodles containing the highest percentage of cassava leaves of 40%.

Pearson's correlation analysis was conducted to estimate the relationships between response variables, i.e., TPC, TCC, and DPPH and FRAP inhibitions (Table S1). The strongest positive correlation value (*r* = 0.97) was obtained for TPC and DPPH inhibitions, while the least (*r* = 0.882) was noticed between TPC and FRAP inhibitions. However, overall, significant (*p* < .01) positive correlations between the TPC, TCC, and DPPH and FRAP inhibitions were observed. Therefore, the findings confirm that cassava could be a good source of dietary phytochemicals, which could enhance the antioxidant properties of the rice analogues.

### 3.8 Morphology of RAF

**FIGURE 3:**
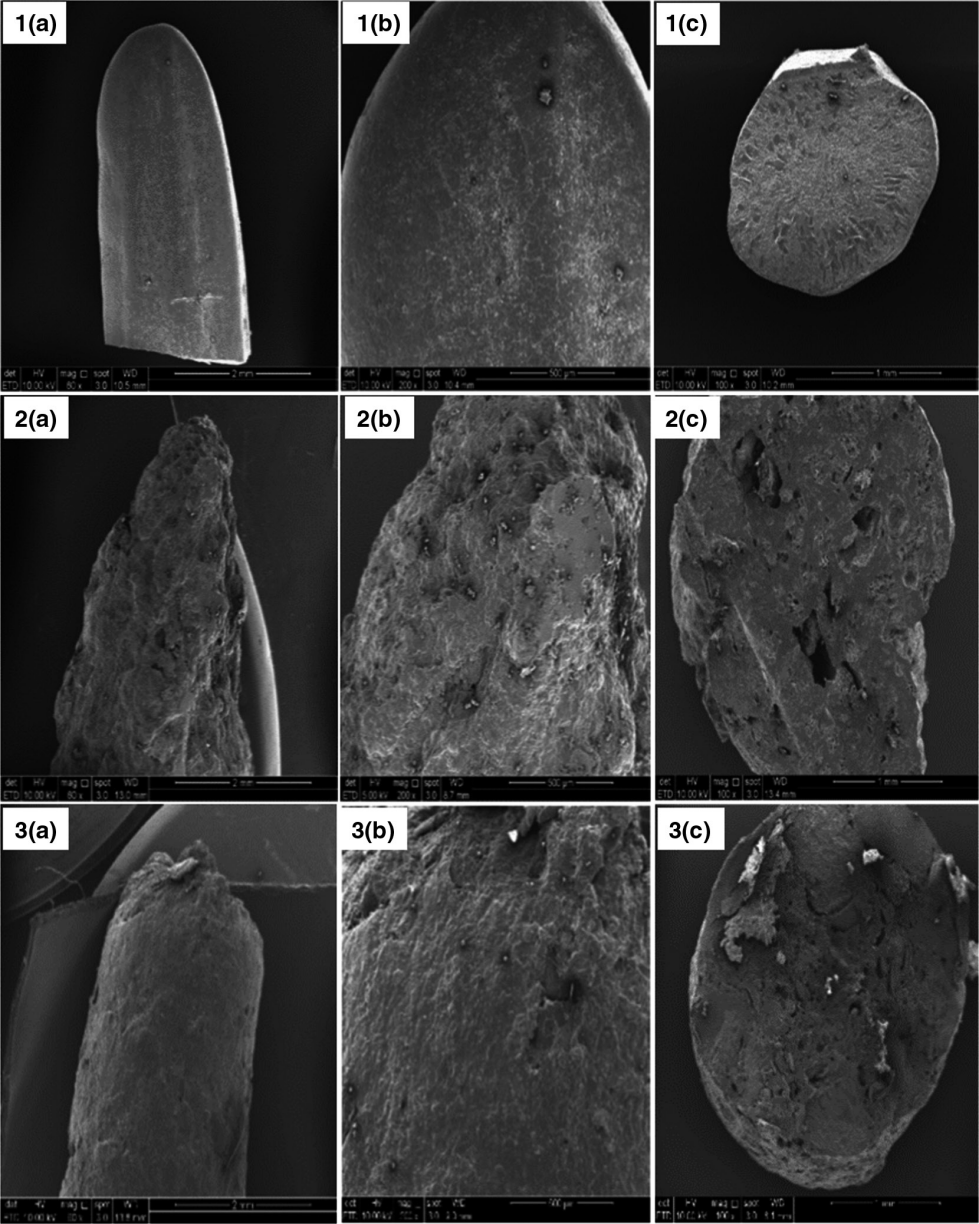
Scanning electron micrographs of the longitudinal section of 1(a) control, 2(a) rice analogue formulation (RAF) 2, and 3(a) RAF 4 at 60× magnification; Surface micrographs of 1(b) control, 2(b) RAF 2, and 3(b) RAF 4 at 200× magnification; Cross‐sections of 1(c) control, 2(c) RAF 2, and 3(c) RAF 4 at 100× magnification; Control = commercial milled rice, RAF 2 = Rice analogue with 5:5 and 20% cassava leaves; RAF 4 = Rice analogue with 0:1; the ratio is expressed in terms of rice flour to modified cassava flour (MOCAF)

Morphological analyses of the control (commercial milled rice), RAF 2 (20% cassava leaves), and RAF 4 (without cassava leaves) were conducted only. Samples of RAF 2 and RAF 4 were selected because they have the highest values of nutrients, proximate analysis (crude protein and fiber, and amylose content), and antioxidants, respectively. Commercial milled rice was used as a control at this stage because the mechanical effects of rice analogue production were to be studied. Based on the FESEM micrographs, the surface of commercial milled rice appeared smoother as compared to those of RAF 2 and RAF 4 (Figure 3). The rough and porous surface of RAF 2 and RAF 4 could be due to uneven mixing during the processing of RAF and the crooked structure of cassava starch due to processing. However, RAF 4 appeared to be smoother as compared to RAF 2, though there were some big pores noticed for RAF 4. Therefore, it is suggested that prolonged mixing could be carried out to improve the appearance of RAF.

From the images of the cross‐section, two distinct layers of bran and endosperm can be observed on the surface of commercial milled rice. Uneven cracks and pores were observed in the cross‐section of RAF 2 and RAF 4, which could be due to starch gelatinization during thermal processing of rice analogue. The cracks were suggested as channels for penetration of water during cooking (Ogawa et al., 2003). Jung et al. (2016) reported a similar microstructure for gelatinized rice grains. The ruptured cell wall can also be observed on the cross‐sectional surface of RAF 4. Ogawa et al. (2003) observed visible cell disruption of cooked rice grain in the cross‐section of rice grain which could be due to gelatinization of starch. During the processing of RAF, MOCAF was mixed with hot water and cooked for a few minutes, which resulted in starch gelatinization of MOCAF.

## 4 CONCLUSION

The study showed that the substitution of rice flour with MOCAF and the addition of cassava leaves had augmented the nutritional and antioxidant properties of cassava‐based rice analogue with a safe level of cyanide. Furthermore, improved functional properties such as swelling power and water absorption capacity were noticed for RAF samples. Conversely, RAF appeared greener and darker compared to control with the addition of cassava leaves. Besides, among all samples, RAF 4 with 20% cassava leaves presented the highest values for TPC, TCC, and DPPH and FRAP inhibitions. This study depicted that cassava‐based RAF has the potential to be a healthy alternative source of food. Nonetheless, the sensory and organoleptic properties of RAF should be evaluated thoroughly to warrant consumer acceptance of this nutritious food product.

## CONFLICT OF INTEREST

The authors declare no conflict of interest.

## ETHICAL APPROVAL

This study does not involve any human or animal testing.

## Supporting information

Supplementary Material

## Data Availability

The data that support the findings of this study are available from the corresponding author, upon reasonable request.
