# Supplementary material for: Formulation and characterization of physicochemical, functional, morphological, and antioxidant properties of cassava‐based rice analogue
Source: Food Sci Nutr. 2022 Feb 21;10(5):1626–37. doi: 10.1002/fsn3.2785 (PMC9094478; doi:10.1002/fsn3.2785)
Supplement: Supplementary file 1 — Supplementary Material [file FSN3-10-1626-s001.docx]

**Supplementary data**

Table S1: Pearson’s correlation coefficient among total phenolic contents (TPC), total chlorophyll contents (TCC), ferric reducing antioxidant power (FRAP), and diphenyl-1-picrylhydrazyl (DPPH) scavenging activity

|  | TPC | DPPH | FRAP | TCC |
| --- | --- | --- | --- | --- |
| TPC | 1 | 0.976 | 0.882 | 0.985 |
| DPPH | 0.976 | 1 | 0.890 | 0.968 |
| FRAP | 0.882 | 0.890 | 1 | 0.912 |
| TCC | 0.985 | 0.968 | 0.912 | 1 |

*Correlation is significant at 0.01 level (2-tailed)


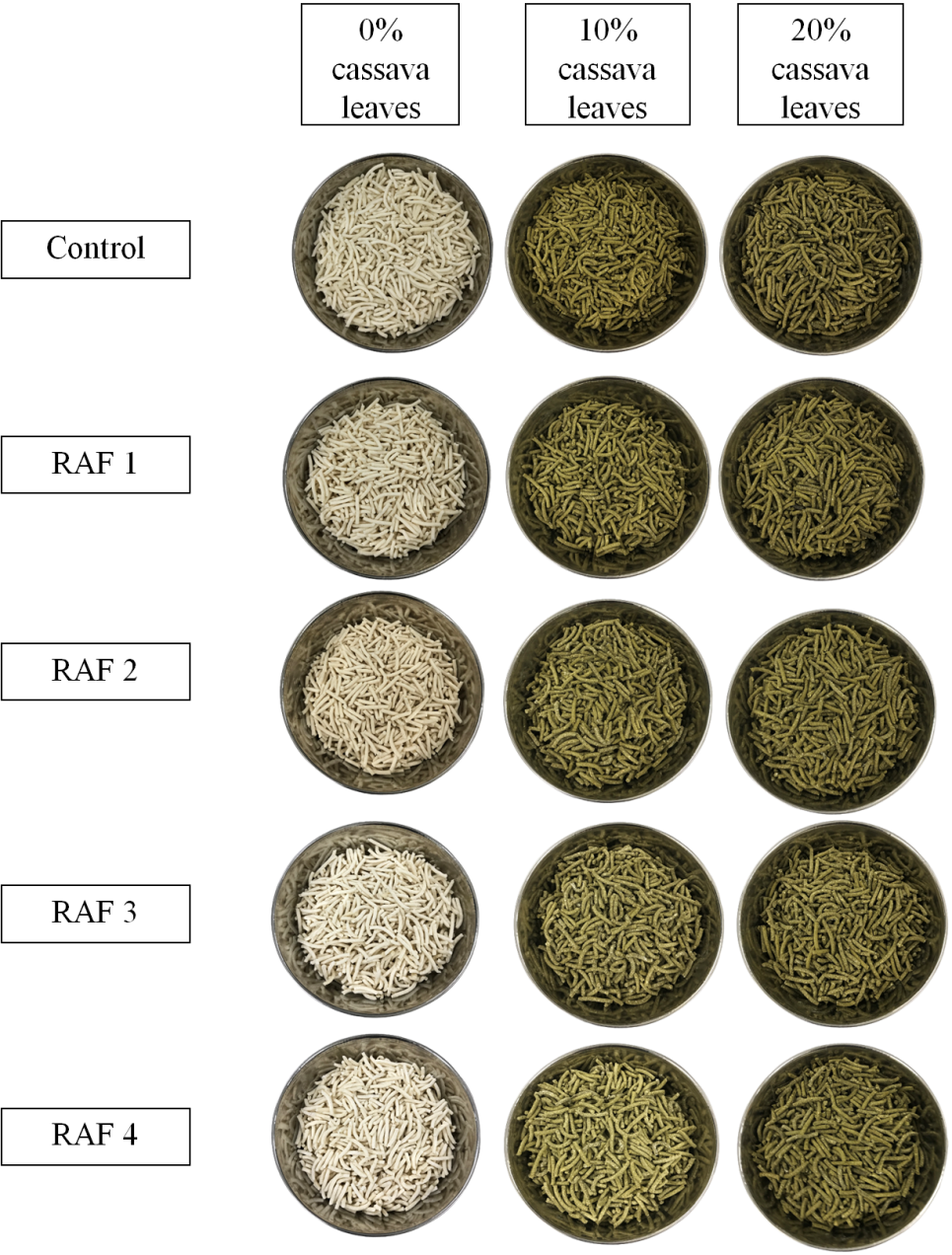


Figure S1:Control = Rice analogue with 1:0 in terms of ratio of rice flour to modified cassava flour (MOCAF) with 0 %, 10 % and 20 % cassava leaves from left to right.

RAF 1 = Rice analogue with 7:3 in term of ratio of rice flour to MOCAF with 0 %, 10 % and 20 % cassava leaves from left to right..

RAF 2 = Rice analogue with 5:5 in term of ratio of rice flour to MOCAF with 0 %, 10 % and 20 % cassava leaves from left to right.

RAF 3 = Rice analogue with 3:7 in term of ratio of rice flour to MOCAF with 0 %, 10 % and 20 % cassava leaves from left to right.

RAF 4 = Rice analogue with 0:1 in term of ratio of rice flour to MOCAF with 0 %, 10 % and 20 % cassava leaves from left to right..
